# Secondary hyperparathyroidism due to chronic kidney disease and access to clinical treatment and parathyroidectomy in Brazil: a nationwide survey

**DOI:** 10.1590/2175-8239-JBN-2024-0158en

**Published:** 2025-02-24

**Authors:** Lauter Eston Pelepenko, Marcelo Giacomini Louça, Tarcísio Fausto, Sergio Gardano Elias Bucharles, Melani Ribeiro Custódio, Leandro Lucca, Fellype de Carvalho Barreto, Aluízio Barbosa Carvalho, Vanda Jorgetti, José Andrade Moura, Rodrigo Bueno de Oliveira

**Affiliations:** 1 Universidade Estadual de Campinas (UNICAMP), Faculdade de Ciências Médicas, Laboratório para o Estudo Mineral e Ósseo em Nefrologia (LEMON), Campinas, SP, Brazil.; 2 Universidade Estadual de Campinas (UNICAMP), Faculdade de Ciências Médicas, Departamento de Clínica Médica (Nefrologia), Campinas, SP, Brazil.; 3 Universidade Federal do Paraná, Departamento de Clínica Médica, Divisão de Nefrologia, Curitiba, PR, Brazil.; 4 Universidade de São Paulo, Faculdade de Medicina, Hospital das Clínicas, Laboratório de Fisiopatologia Renal, São Paulo, SP, Brazil.; 5 Universidade de São Paulo, Faculdade de Medicina, Hospital de Clínicas, Ribeirão Preto, SP, Brazil.; 6 Universidade Federal de São Paulo, Departamento de Medicina, Divisão de Nefrologia, São Paulo, SP, Brazil.; 7 Escola Bahiana de Medicina e Saúde Pública, Salvador, BA, Brazil.

**Keywords:** Hyperparathyroidism, Secondary, Parathyroidectomy, Renal Dialysis, Hemodialysis Units, Hospital, Renal Insufficiency, Chronic

## Abstract

**Introduction:**

Chronic kidney disease (CKD) may lead to secondary hyperparathyroidism (SHP) and its treatment is based on the control of hyperphosphatemia, hypocalcemia, and serum parathormone hormone levels (PTH) levels. Despite the advances in SHP treatment, therapeutic failure is frequent and CKD patients on dialysis require parathyroidectomy (PTx).

**Aim:**

To update the 2011 survey, estimate the current prevalence of SHP in Brazilian dialysis centers, verify access to drugs, and identify obstacles to performing PTx.

**Methods:**

A questionnaire was sent to active dialysis facilities. The results were compiled and statistically compared (p < 0.05).

**Results:**

A total of 114 facilities successfully responded to the questionnaire, most of them in the Southeast region. Approximately 9% of the individuals (23,535) had serum PTH levels measurements above 1,000 pg/mL (10.7% were reported in the 2011 survey). A considerable number of the reported difficulties indicated limited availability of pivotal medications for SHP management and the associated complications. Of note, only 2.7% of the individuals were submitted to PTx. For those with PTx indication, the waiting time for the procedure was over two years in 28% of the cases. The main barriers to performing PTx were reported to be the long waiting time for PTx, the shortage of head and neck surgeons, and the lack of ward beds for hospital admissions.

**Conclusion:**

Some aspects have improved since 2011. However, SHP remains highly prevalent in Brazil, and a significant number of individuals do not have access to PTx or experience long waiting times for this surgical procedure while facing substantial difficulties in obtaining clinical treatment.

## Introduction

Chronic kidney disease (CKD) affects millions of people and is a major health challenge worldwide^1^. People with CKD have a high morbidity and mortality rate^2^, a significant proportion of which is attributed to mineral bone disorder (MBD)^3^, a condition involving biochemical and hormonal disturbances, vascular calcification, and bone diseases^4^. A well-defined condition within the spectrum of MBD is secondary hyperparathyroidism (SHP)^5^. With CKD progression, there is reduced synthesis of calcitriol, a trend towards low serum calcium levels, with increased serum of phosphorus, fibroblast growth factor 23 (FGF-23), resulting in a progressive increase in serum parathyroid hormone levels (PTH)^6^ in many patients^7,8,9^. These alterations were also associated with vascular calcification and bone fractures and are associated with an increased mortality rate^10,11,12^.

SHP treatment is traditionally based on control of hyperphosphatemia including dietary restriction, dialysis, administration of P-binder drugs, reversal of hypocalcemia with administration of calcium salts and calcitriol, and control of the PTH levels with calcitriol, selective vitamin D-receptor activators and calcimimetics^13,14,15^. Despite these advances in the treatment of SHP, therapeutic failure is frequent^16^, with an estimated 5-30% of CKD patients on dialysis eventually undergoing parathyroidectomy (PTx)^17,18,19,20^ due to an inadequate response to medical therapy^21^; worryingly, this burden may increase with dialysis time^22,23,24,25^.

Taken together with these data, a 2017 meta-analysis indicated the clinically significant beneficial effect of PTx on all-cause and cardiovascular mortality in CKD patients with SHP^26^ and is also advised, considering certain conditions, by the Kidney Disease Improving Global Outcomes (KDIGO) MBD-CKD 2017 update^27^, and by other publications involving patients with severe SHP^18,19,20,28^. However, given the observational nature of most of the analyzed studies, the choice between clinical and surgical treatments is still under debate, especially when considering different stages of SHP^29^. Thus, a randomized controlled trial comparing surgery with medical therapy is required.

According to the 2023 United States Renal Data System (USRDS), the prevalence of dialysis in Brazil is 716 per million (ranking in the 21^st^ position) (https://usrds-adr.niddk.nih.gov/2023/end-stage-renal-disease/11-international-comparisons). The Brazilian Dialysis Survey 2022^30^ identified 153,831 patients receiving dialysis treatment, spread across 872 active clinics in the country indicating that the absolute number and prevalence rate of patients on chronic dialysis continues to increase, as corroborated by a previous census regarding these rates^31^. Evidence has shown that a significant proportion of patients in Brazil who develop SHP do not have access to full clinical or surgical treatment. A key point would be to generate data to support public and private healthcare providers and inform government officials about the importance of improving public health policies for the treatment of Mineral and Bone Disorder in Chronic Kidney Disease (MBD-CKD)^32^. The quality and continuity of care have a direct impact on the prevention of these bone complications; thus the risk of discontinuity must be taken into account by both governmental and private centers.

In 2011, the first Brazilian survey on the surgical treatment (PTx) of SHP was reported^33^. After thirteen years, important events have affected the treatment of CKD in Brazil, including the introduction of new national MBD-CKD guidelines, the establishment of international dialysis companies in the country (DaVita, Fresenius Medical Care and Diaverum), and changes caused by the COVID-19 pandemic. Altogether, these factors influenced the MBD-CKD experts’ perception of the challenges in managing SHP patients in Brazil.

The purpose of this study is to update the 2011 census^33^, estimate the current prevalence of severe SHP among Brazilian dialysis centers, evaluate the access to drugs for SHP treatment, and identify the main obstacles to performing PTx.

## Methods

This study is a cross-sectional national survey. From April to June 2024, the Committee on Mineral and Bone Disorder in Chronic Kidney Disease of the Brazilian Society of Nephrology (SBN) sent an online questionnaire to the technical Directors of the dialysis units in Brazil. The questions addressed the diagnosis and management of MBD and focused on SHP. The centers were invited to report information about SHP related to the first semester of 2024 (reference, April/2024).

The questionnaire (available in Portuguese at https://shorturl.at/7A31n) consisted of 21 questions on the geographic location of the facility, the categorization of individuals according to serum PTH levels (<100 pg/mL, >600 pg/mL and >1,000 pg/mL), and the difficulties in clinical and surgical management of SHP. An open 6-point Likert scale was used for data collection, as indicated in the questionnaire structure. The Likert scale is commonly used in questionnaires to record opinion polls where the respondents specify their level of agreement with a proposed statement. Respondents were guaranteed that they could choose their answers freely and spontaneously, according to their personal experiences and local conditions in which they work.

According to the Brazilian Dialysis Survey 2022, there are 872 active dialysis clinics registered with the Brazilian Society of Nephrology^30^. All units were contacted by email and/or phone and invited to complete the survey. An electronic alert was issued every 15 days between April and June aiming to increase adherence. The authors did not get access to patient primary or secondary data, relying solely on the data provided by the respondent facilities.

### Statistical Analysis

The data from the respondent units were described in absolute values and percentages. Graphical representations of mean and standard deviation were developed using GraphPad Prism version 10 (Prism, USA). Data normality was assessed using Shapiro-Wilk test and comparisons were obtained using ANOVA and Kruskal-Wallis, according to the data normality obtained.

## Results

**Figure 1:**
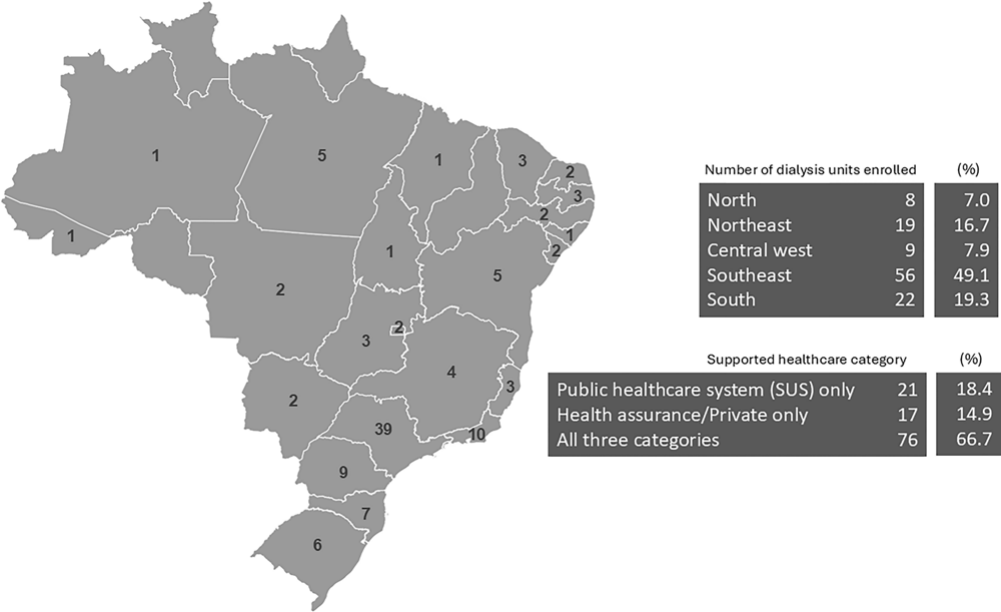
Distribution and percentage by state and region of the healthcare category (Brazilian Public Healthcare System [SUS], health assurance, or private).

A total of 114 facilities (13%) successfully returned the questionnaire. All Brazilian states, except Roraima, Amapá, Rondônia and Piauí states were represented by at least one responding facility. Figure 1 indicates the nationwide percent distribution by state, region, and facility financial agreements, indicating that almost half of the respondent facilities are in the Southeast region, mainly in the city of São Paulo.

Note – that the same facility may enroll in more than one healthcare funding.

The Brazilian Public Healthcare System (SUS) is the primary funding source for most respondent facilities, which also accept health insurance contracts and out-of-pocket private enrollments. Table 1 presents data from these facilities, encompassing 23,535 individuals, representing 15.3% of the dialysis population in Brazil, classified according to their serum PTH levels (<100 pg/mL, >600 pg/mL, and >1,000 pg/mL). On average, about 9% of the individuals had serum PTH levels >1,000 pg/mL. This number increases to about 20% considering serum PTH levels >600 pg/mL. In contrast, only approximately 3% had submitted to PTx. The North region had significantly less individuals in comparison with the Southeast region (p < 0.05).

**Table 1:** Declared number of individuals by region, serum pth levels range (<100 pg/ml, >600 pg/ml, and >1,000 pg/ml), and the prevalence of parathyroidectomy

Region	N (%)		N (%) serum PTH levels			N (%) submitted to parathyroidectomy
			<100 pg/ml	>600 pg/ml	>1,000 pg/ml	
North	875^b^ (3.7)		277 (31.7)	242 (27.7)	193 (22.1)	90 (10.3)
Northeast	4,406^ab^ (18.7)		635 (14.4)	1,037 (23.5)	447 (10.1)	85 (1.9)
Central west	1,763^ab^ (7.5)		220 (12.5)	322 (18.3)	209 (11.9)	25 (1.4)
Southeast	12,849ª (54.6)		2,346 (18.3)	2,524 (19.6)	1,006 (7.8)	333 (2.6)
South	3,642^ab^ (15.5)		912 (25.0)	510 (14.0)	232 (6.4)	95 (2.6)
**Total**	**23,535 (100.0)**		**4,390 (18.7)**	**4,635 (19.7)**	**2,087 (8.9)**	**628 (2.7)**

Note: Different letters (a, b) indicate significant differences between regions.

**Figure 2:**
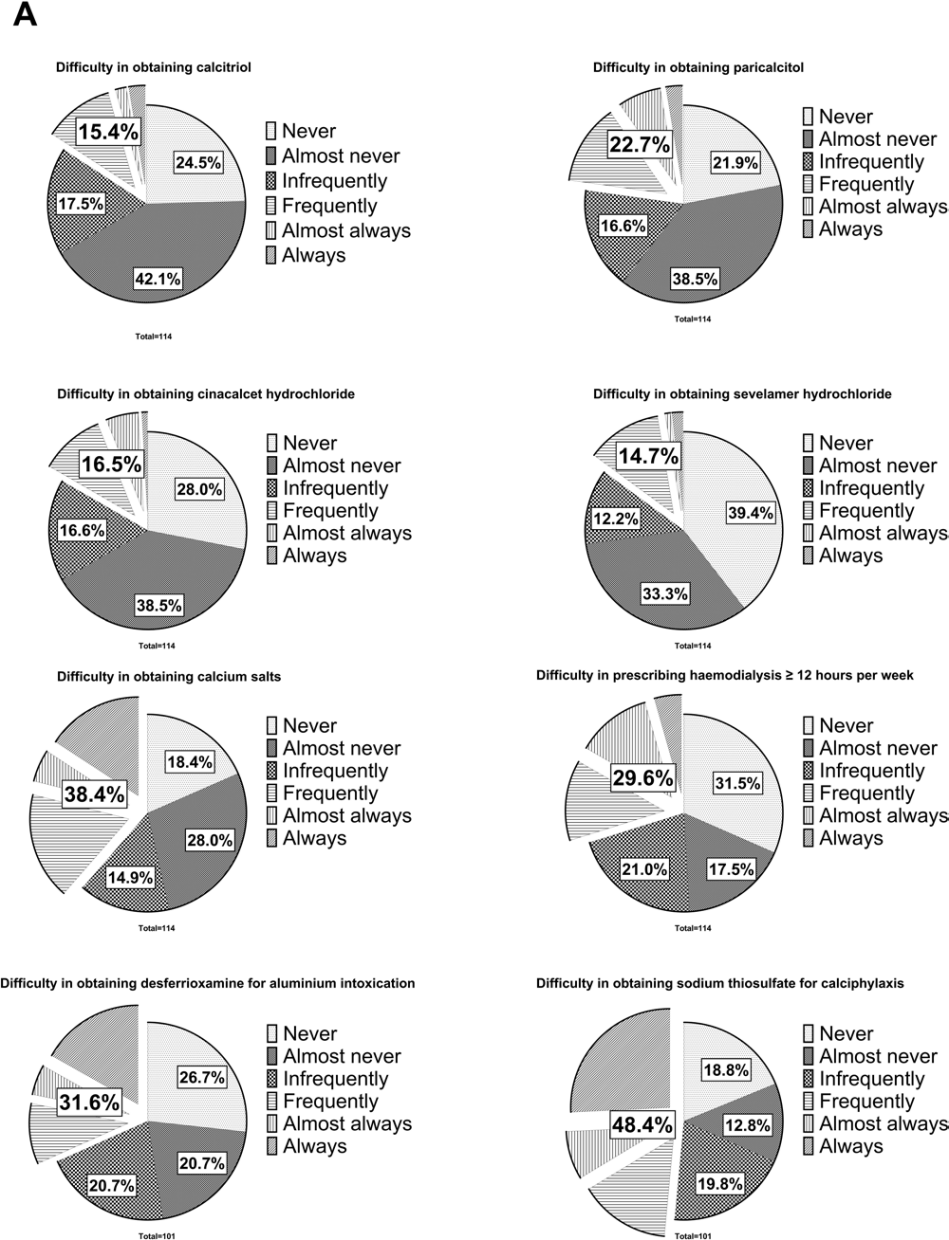
Difficulties in obtaining medications and prescribing hemodialysis for the management of these individuals; captions are provided clockwise.

**Figure 3:**
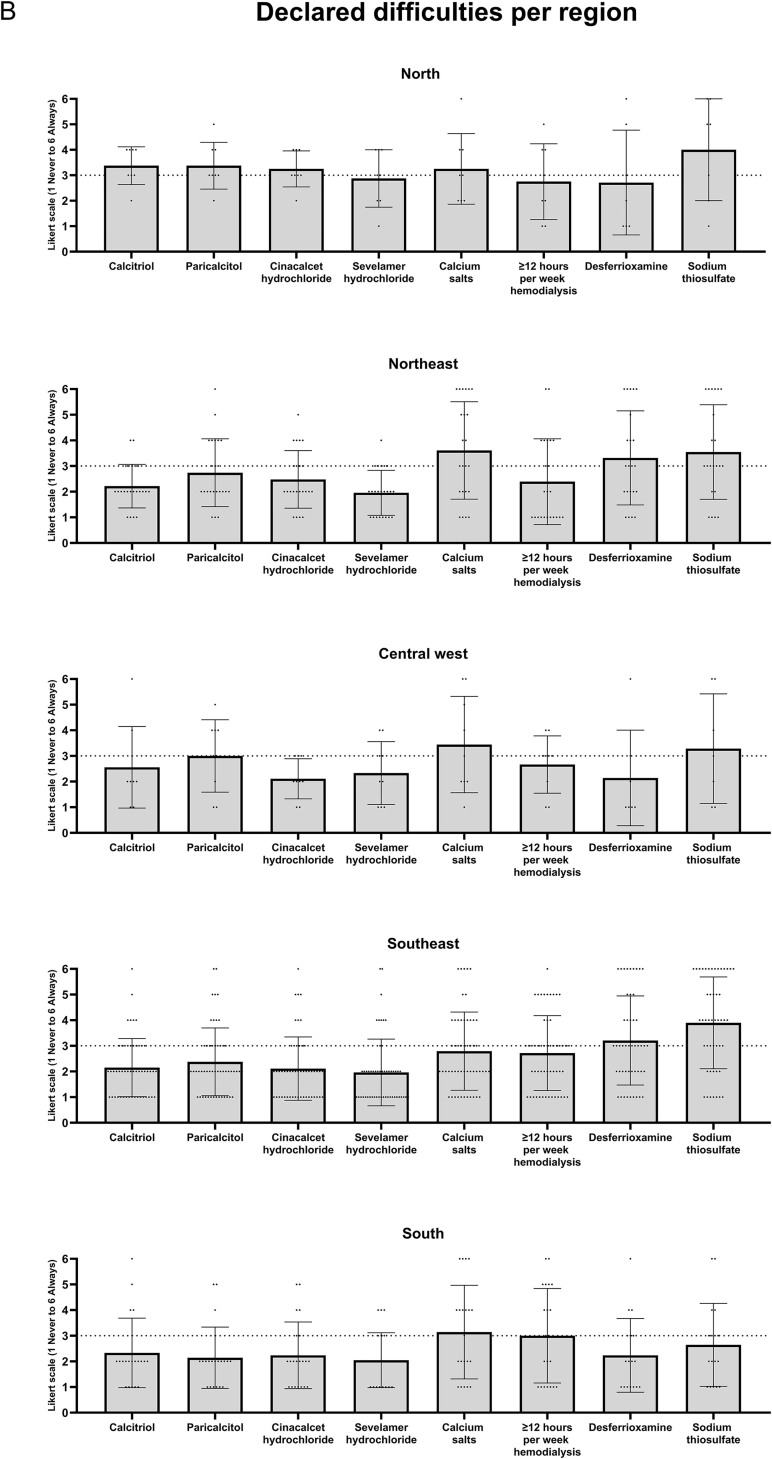
Data classified by region. Each dot represents one respondent facility and the cutoff line at level “three” of the Likert scale (“infrequently”) is shown by a dashed line.

Figure 2 represents the reported difficulties in obtaining medications and achieving hemodialysis weekly time treatment ≥12h for patient management, and Figure 3 shows these difficulties by region/medication. Calcitriol, paricalcitol, cinacalcet hydrochloride, and sevelamer hydrochloride were reported as easy to obtain (from never to rarely with difficulty). Worryingly, difficulties in obtaining these were reported by 14 to 23% of the facilities, highlighting differences in how these difficulties are managed. A crucial observation of the data is the difficulties reported in obtaining calcium salts by almost 40% of the facilities; besides, around 30% of them also indicated difficulties in prescribing hemodialysis ≥12 hours per week and in obtaining desferrioxamine for aluminum intoxication treatment. Finally, the greatest difficulty reported by almost half of the facilities was in obtaining sodium thiosulfate for calciphylaxis management. Of note, except for sodium thiosulfate, all these drugs are provided by the SUS.

Analyzed by region (Figure 3), calcitriol, paricalcitol, cinacalcet hydrochloride, sevelamer hydrochloride, and sodium thiosulfate were declared to be on average “frequently” to “always” difficult to obtain in the North region, whereas calcium salts and desferrioxamine were similarly difficult to obtain in the Northeast region. Scheduling above 12 hours of hemodialysis per week was declared to be on average “frequently” to “always” achievable in the South region.

**Figure 4:**
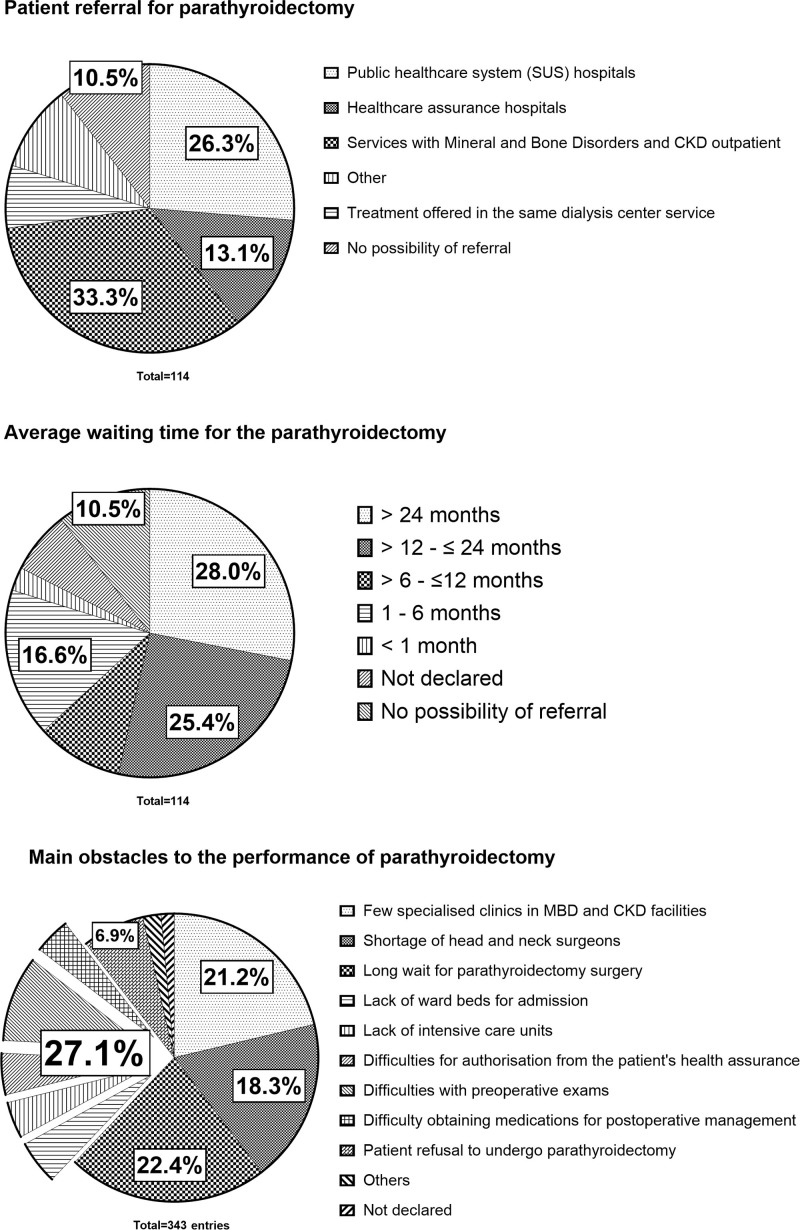
Responses regarding the referral options for parathyroidectomy (PTx), the average waiting time, and the main obstacles to the performance of PTx; captions are provided clockwise.

The data regarding the referral options for PTx, the waiting time, and the main obstacles to the performance of PTx are graphically represented in Figure 4. The three main referrals for PTx are public services with MBD-CKD specialized staff, followed by the SUS hospitals, and healthcare assurance hospitals. Worryingly, the waiting time for the procedure was mostly over two years and uncommonly performed under six months of waiting; also, the shortage of head and neck surgeons, and the lack ward beds for hospital admissions were declared as important obstacles. Difficulties in authorizing the procedure within the healthcare assurance contract operator accounted for around 27% of the difficulties. In addition, preoperative exams, and medication obtention for postoperative management were declared barriers. No possibility of referral accounts for 10%.

Comparison between data from the 2011 study and this 2024 update is shown in Table 2. Even though a direct statistical comparison is beyond the aim of the present update, the absolute number – and percentage – of patients with PTH > 1,000 pg/mL and those with indication or who underwent PTx remained similar representing a small improvement during the last thirteen years of management of this condition.

**Table 2:** Comaparison between selected data from the 2011^33^ survey and the 2024 update

Study	Total number of individuals	Total number (%) of patients with PTH > 1,000 pg/ml	Units (%) with waiting time of 12 to 24 months for PTx	Units (%) declaring shortage of head and neck surgeons
2011	32,264	3,463 (10.7)	19 (8)	64 (28)
2024	23,535	2,087 (8.9)	29 (25)	63 (18)

## Discussion

This study aimed to update and deepen the 2011 survey^33^. The prevalence rate of severe SHP was reported to be around 9%, considering PTH levels >1,000 pg/mL, a situation that requires PTx, and can reach 20% considering serum PTH levels >600 pg/mL. Further evidence that surgical intervention is the best treatment option was recently reported^16^ and indicated for CKD patients on dialysis^17–20^. If the 800 pg/mL cut-off point were adopted, as recommended by the Clinical Practice MBD-CKD Brazilian Guidelines, the prevalence of SHP would be higher. It is also noteworthy that a significant percentage of individuals with PTH up to 500 pg/mL do not fully respond to medical therapy either^34^, which would further increase the number of individuals requiring PTx surgery. It is remarkable that the number of patients submitted to PTx represents 2.7% of the individuals; however, this information has not been reported in the previous survey. In our opinion, this information is worrying. First, it is necessary to emphasize that medications, even simpler ones such as calcium salts, and adequate conditions for prescribing the appropriate time of dialysis therapy are efficient and low-cost ways to prevent and delay the progression of MBD-CKD. Overcoming these obstacles is an essential element in changing this reality.

Being a moderately complex surgical procedure, PTx could be performed in secondary-care hospitals staffed with nephrologists (dialysis support) and head and neck surgeons^33^. In these 2024 results, the two main obstacles – few specialized MBD-CKD facilities that support this procedure and a shortage of head and neck surgeons – remain significant challenges. However, nephrologists are fully able to manage MBD-CKD patients.

Around 90% of patients undergoing PTx develop the ‘hunger bone’ syndrome during the postoperative period^35^, requiring high amounts of calcium salts and calcitriol for several weeks. Although most facilities receive these medications from the Special Medications Program of the SUS, bureaucratic constraints can still lead to delayed dispensation of insufficient amounts. This issue was reported as “frequent” by 17.2% of facilities for calcium salts and 8.6% for calcitriol. Consequently, facility management may need to redirect these medications from patients with hypercalcemia and/or hyperphosphatemia.

According to the 2023 Medical Demography in Brazil (https://amb.org.br/wp-content/uploads/ 2023/02/DemografiaMedica2023_8fev-1.pdf), the number of head and neck surgeons has increased to 1,403 professionals. The majority reside in Sao Paulo, as previously indicated^33^ and perhaps the crucial concern regarding technical, political, economic, and organizational challenges that hinder collaboration among public health managers, dialysis units, nephrologists, head and neck surgeons, and centers performing PTx are still relevant in 2024.

The previously reported obstacle of preoperative exams (~39%)^33^ for performing PTx does not appear to be the main issue in 2024, as indicated by less than 5% of respondents in this update. The observed concentration of facilities in the Southeast region might have influenced this association. Conversely, in 2024, the declared obstacle of few specialized MBD-CKD facilities that supports PTx associated with the declared shortage of head and neck surgeons seemed to be the main two issues. The lack of intensive care units and ward beds was also cited as a problem, as in 2011^33^.

This survey update has limitations. While this study aimed to update the 2011 survey^33^, the response rate was lower, with 1.9 times more dialysis units (226 in that study including 32,264 individuals) participating in the earlier survey. As a result, the data presented here should be interpreted with caution, especially since the number of respondents units was reduced to 114, being mostly from the city of São Paulo. This census did not aim to analyze the causes and factors involved in the difficulties related to access to clinical and surgical treatment for SHP. Another aspect that draws attention is that in 2011 the average number of patients was 143 per dialysis center and in 2024, it rose to 206 per center, an increase of 44%. The impact of this increase on MBD-CKD could not be captured by this survey but deserves consideration as a possible factor associated with the observed results.

Even though the questionnaire did not focus on the continuity of medication supply, it is reasonable to speculate that medication interruptions may play a negative role in the control of SHP. Finally, this study provides a national survey with representative data from the dialysis population across all regions of the country and collectively reveals the challenges in accessing clinical and surgical treatment for SHP.

## Conclusion

Some aspects of SHP in patients under dialysis have improved since 2011. However, the condition remains highly prevalent in Brazil, and a significant number of individuals do not have access to PTx or need to wait for a long time for this surgical procedure while facing substantial difficulties in obtaining clinical treatment. While government efforts to provide access to clinical and surgical treatment to patients are commendable, there might be room for system improvements. Hopefully, these data can potentially support enhancements in the healthcare system for these patients through better public healthcare. More efficient collaboration between nephrologists and surgeons, and both public and private healthcare providers, are urgently required to change this reality.

## Data Availability

All data generated or analyzed during this study are included in this article.
