# Supplementary material for: Secondary hyperparathyroidism due to chronic kidney disease and access to clinical treatment and parathyroidectomy in Brazil: a nationwide survey
Source: J Bras Nefrol. 2025 Feb 24;47(2):e20240158. doi: 10.1590/2175-8239-JBN-2024-0158en (PMC11855617; doi:10.1590/2175-8239-JBN-2024-0158en)
Supplement: Translation

# Translation: Hiperparatireoidismo secundário devido à doença renal crônica e acesso ao tratamento clínico e à paratireoidectomia no Brasil: uma pesquisa nacional

**DOI:** 10.1590/2175-8239-JBN-2024-0158pt

**Authors:** Lauter Eston Pelepenko, Marcelo Giacomini Louça, Tarcísio Fausto, Sergio Gardano Elias Bucharles, Melani Ribeiro Custódio, Leandro Lucca, Fellype de Carvalho Barreto, Aluízio Barbosa Carvalho, Vanda Jorgetti, José Andrade Moura, Rodrigo Bueno de Oliveira

**Affiliations:** 1 Universidade Estadual de Campinas (UNICAMP), Faculdade de Ciências Médicas, Laboratório para o Estudo Mineral e Ósseo em Nefrologia (LEMON), Campinas, SP, Brasil.; 2 Universidade Estadual de Campinas (UNICAMP), Faculdade de Ciências Médicas, Departamento de Clínica Médica (Nefrologia), Campinas, SP, Brasil.; 3 Universidade Federal do Paraná, Departamento de Clínica Médica, Divisão de Nefrologia, Curitiba, PR, Brasil.; 4 Universidade de São Paulo, Faculdade de Medicina, Hospital das Clínicas, Laboratório de Fisiopatologia Renal, São Paulo, SP, Brasil.; 5 Universidade de São Paulo, Faculdade de Medicina, Hospital de Clínicas, Ribeirão Preto, SP, Brasil.; 6 Universidade Federal de São Paulo, Departamento de Medicina, Divisão de Nefrologia, São Paulo, SP, Brasil.; 7 Escola Bahiana de Medicina e Saúde Pública, Salvador, BA, Brasil.

**Keywords:** Hiperparatireoidismo Secundário, Paratireoidectomia, Diálise Renal; Unidades Hospitalares de Hemodiálise, Insuficiência Renal Crônica

## Abstract

**Introdução:**

A doença renal crônica (DRC) pode levar ao hiperparatireoidismo secundário (HPTS) e seu tratamento baseia-se no controle da hiperfosfatemia, hipocalcemia e níveis séricos de paratormônio (PTH). Apesar dos avanços no tratamento do HPTS, a falha terapêutica é frequente, e pacientes com DRC em diálise demandam paratireoidectomia (PTx).

**Objetivo:**

Atualizar a pesquisa de 2011, estimar a prevalência atual de HPTS nos centros de diálise brasileiros, verificar acesso a medicamentos e identificar obstáculos para a realização de PTx.

**Métodos:**

Questionário enviado às unidades de diálise ativas. Os resultados foram compilados e comparados estatisticamente (p < 0,05).

**Resultados:**

114 unidades responderam com sucesso ao questionário, a maioria da região sudeste. Aproximadamente 9% dos indivíduos (23.535) apresentaram níveis séricos de PTH acima de 1.000 pg/mL (10,7% foram relatados em 2011). Um número considerável das dificuldades relatadas indicou disponibilidade limitada de medicamentos essenciais para o manejo do HPTS e complicações associadas. Ressalta-se que apenas 2,7% dos indivíduos foram submetidos à PTx. Para aqueles com indicação de PTx, o tempo de espera pelo procedimento foi superior a dois anos em 28% dos casos. As principais barreiras à realização da PTx foram relatadas como o longo tempo de espera pelo procedimento, a escassez de cirurgiões de cabeça e pescoço e a falta de leitos para internações hospitalares.

**Conclusão:**

Alguns aspectos melhoraram desde 2011. Entretanto, o HPTS continua altamente prevalente no Brasil, e um número significativo de indivíduos não tem acesso à PTx ou enfrenta longos períodos de espera por esse procedimento cirúrgico, além de dificuldades substanciais para obter tratamento clínico.

## Introdução

A doença renal crônica (DRC) afeta milhões de pessoas e é um grande desafio para a saúde mundial^1^. Pessoas com DRC apresentam altas taxas de morbidade e mortalidade^2^, com uma proporção significativa sendo atribuída ao distúrbio mineral e ósseo (DMO)^3^, uma condição que envolve distúrbios bioquímicos e hormonais, calcificação vascular e doenças ósseas^4^. Uma condição bem definida dentro do espectro do DMO é o hiperparatireoidismo secundário (HPTS)^5^. Com a progressão da DRC, ocorre uma síntese reduzida de calcitriol, uma tendência a baixos níveis séricos de cálcio, com aumento sérico de fósforo, fator de crescimento de fibroblastos 23 (FGF-23), resultando em um aumento progressivo dos níveis séricos de paratormônio (PTH)^6^ em um grande número de pacientes^7–9^. Essas alterações também foram associadas à calcificação vascular e fraturas ósseas e estão relacionadas ao aumento da taxa de mortalidade^10–12^.

O tratamento do HPTS é tradicionalmente baseado no controle da hiperfosfatemia, incluindo restrição dietética, diálise e administração de medicamentos quelantes de P, reversão da hipocalcemia com a administração de sais de cálcio e calcitriol e controle dos níveis de PTH com calcitriol, ativadores seletivos do receptor de vitamina D e calcimiméticos^13–15^. Apesar desses avanços no tratamento do HPTS, a falha terapêutica é frequente^16^, estimando-se que de 5% a 30% dos pacientes com DRC em diálise eventualmente sejam submetidos à paratireoidectomia (PTx)^17–20^ devido a uma resposta inadequada à terapia médica^21^; de forma preocupante, esse ônus pode aumentar com o tempo de diálise^22–25^.

Juntamente com esses dados, uma metanálise de 2017 indicou o efeito benéfico clinicamente significativo da PTx na mortalidade cardiovascular e por todas as causas em pacientes com DRC com HPTS^26^. Além disso, o procedimento também é recomendado, considerando determinadas condições, pela atualização de 2017 do *Kidney Disease: Improving Global Outcomes* (KDIGO) para DMO-DRC^27^ e por outras publicações envolvendo pacientes com HPTS grave^18–20,28^. No entanto, devido à natureza observacional da maioria dos estudos analisados, a escolha entre os tratamentos clínico e cirúrgico ainda é objeto de debate, especialmente ao considerar diferentes estágios do HPTS^29^. É necessário um ensaio clínico randomizado comparando a cirurgia com a terapia medicamentosa.

De acordo com o *United States Renal Data System* (USRDS) de 2023, a prevalência de diálise no Brasil é de 716 por milhão (ocupando a 21ª posição) (https://usrds-adr.niddk.nih.gov/2023/end-stage-renal-disease/11-international-comparisons). O Censo Brasileiro de Diálise 2022^30^ identificou 153.831 pacientes em tratamento dialítico, distribuídos em 872 clínicas ativas no país, indicando que o número absoluto e a taxa de prevalência de pacientes em diálise crônica continuam a aumentar, conforme corroborado por um censo anterior sobre essas taxas^31^. Evidências têm demonstrado que, no Brasil, uma proporção significativa de pacientes que desenvolvem HPTS não tem acesso a um tratamento clínico ou cirúrgico completo. Um ponto fundamental seria gerar dados para apoiar os profissionais de saúde públicos e privados e informar as autoridades governamentais sobre a importância de melhorar as políticas públicas de saúde para o tratamento do Distúrbio Mineral e Ósseo na Doença Renal Crônica (DMO-DRC)^32^. A qualidade e a continuidade do atendimento exercem um impacto direto na prevenção dessas complicações ósseas, de modo que o risco de descontinuidade deve ser considerado tanto pelos centros governamentais quanto privados.

Em 2011, foi relatado o primeiro levantamento brasileiro sobre o tratamento cirúrgico (PTx) do HPTS^33^. Após treze anos, eventos importantes impactaram o tratamento da DRC no Brasil, incluindo a introdução de novas diretrizes nacionais de DMO-DRC, o estabelecimento de empresas internacionais de diálise no país (*DaVita*, *Fresenius Medical Care* e *Diaverum*) e as mudanças causadas pela pandemia de COVID-19. Esses fatores, em conjunto, influenciam a percepção dos especialistas em DMO-DRC sobre os desafios no manejo de pacientes com HPTS no Brasil.

O objetivo deste estudo é atualizar o censo de 2011^33^, estimar a prevalência atual de HPTS grave entre os centros de diálise brasileiros, avaliar o acesso a medicamentos para o tratamento do HPTS e identificar os principais obstáculos para a realização de PTx.

## Métodos

Este estudo é uma pesquisa nacional de corte transversal. De abril a junho de 2024, o Comitê de Distúrbios Minerais e Ósseos na Doença Renal Crônica da Sociedade Brasileira de Nefrologia (SBN) enviou um questionário online aos diretores técnicos das unidades de diálise do Brasil. As questões abordavam o diagnóstico e o manejo do DMO, com foco no HPTS. Os centros foram convidados a relatar informações sobre HPTS relacionadas ao primeiro semestre de 2024 (referência, abril/2024).

O questionário (disponível em português em https://shorturl.at/7A31n) consistiu em 21 questões sobre a localização geográfica da unidade, a categorização dos indivíduos de acordo com os níveis séricos de PTH (<100 pg/mL, >600 pg/mL e >1.000 pg/mL) e as dificuldades no manejo clínico e cirúrgico do HPTS. Uma escala Likert aberta de 6 pontos foi utilizada para a coleta de dados, conforme indicado na estrutura do questionário. A escala Likert é comumente usada em questionários para registrar pesquisas de opinião, onde os respondentes especificam seu nível de concordância com uma afirmação proposta. Foi garantido aos entrevistados que eles poderiam escolher suas respostas de forma livre e espontânea, de acordo com suas experiências pessoais e condições locais em que trabalham.

De acordo com o Censo Brasileiro de Diálise 2022, existem 872 clínicas de diálise ativas registradas na Sociedade Brasileira de Nefrologia^30^. Todas as unidades foram contatadas por e-mail e/ou telefone e convidadas a responder à pesquisa. Um alerta eletrônico foi emitido a cada 15 dias entre abril e junho, com o objetivo de aumentar a adesão. Os autores não tiveram acesso aos dados primários ou secundários dos pacientes, confiando exclusivamente nos dados fornecidos pelas unidades respondentes.

### Análise Estatística

Os dados das unidades respondentes foram descritos em valores absolutos e porcentagens. As representações gráficas de média e desvio padrão foram desenvolvidas utilizando o *GraphPad Prism* versão 10 (Prism, EUA). A normalidade dos dados foi avaliada pelo teste de Shapiro-Wilk e as comparações foram obtidas por meio de ANOVA e Kruskal-Wallis, de acordo com a normalidade dos dados obtida.

## Resultados

Um total de 114 unidades (13%) retornaram com sucesso o questionário. Todos os estados brasileiros, exceto Roraima, Amapá, Rondônia e Piauí, foram representados por pelo menos uma unidade respondente. A Figura 1 indica a distribuição percentual nacional por estado, região e acordos financeiros das instalações, mostrando que quase metade das unidades respondentes está localizada na região Sudeste, principalmente na cidade de São Paulo.

**Figura 1:** Distribuição e porcentagem por estado e região da categoria de assistência médica (Sistema Único de Saúde [SUS], plano de saúde ou atendimento particular).

Nota – Ressalte-se que uma mesma unidade pode se inscrever em mais de uma modalidade de financiamento de saúde.

O Sistema Único de Saúde (SUS) é a principal fonte de financiamento para a maioria das unidades participantes, que também aceitam contratos de planos de saúde e inscrições particulares custeadas pelo próprio paciente. A Tabela 1 apresenta os dados dessas unidades, abrangendo 23.535 indivíduos, representando 15,3% da população em diálise no Brasil, classificados de acordo com seus níveis séricos de PTH (<100 pg/mL, >600 pg/mL e >1.000 pg/mL). Em média, cerca de 9% dos indivíduos apresentaram níveis séricos de PTH >1.000 pg/mL. Esse número aumenta para cerca de 20%, considerando níveis séricos de PTH > 600 pg/mL. Em contrapartida, apenas aproximadamente 3% foram submetidos a PTx. A região Norte apresentou um número significativamente menor de indivíduos em comparação com a região Sudeste (p < 0,05).

**Tabela 1:** Número declarado de indivíduos por região, faixa de níveis séricos de pth (<100 pg/ml, >600 pg/ml e >1.000 pg/ml) e prevalência de paratiroidectomia

Região	N (%)		N (%) níveis séricos de PTH			N (%) submetidos à paratireoidectomia
			<100 pg/ml	>600 pg/ml	>1,000 pg/ml	
Nordeste	875^b^ (3,7)		277 (31,7)	242 (27,7)	193 (22,1)	90 (10,3)
Centro-Oeste	4.406^ab^ (18,7)		635 (14,4)	1.037 (23,5)	447 (10,1)	85 (1,9)
Sudeste	1.763^ab^ (7,5)		220 (12,5)	322 (18,3)	209 (11,9)	25 (1,4)
Sul	12.849ª (54,6)		2.346 (18,3)	2.524 (19,6)	1.006 (7,8)	333 (2,6)
South	3.642^ab^ (15,5)		912 (25,0)	510 (14,0)	232 (6,4)	95 (2,6)
**Total**	**23.535 (100,0)**		**4.390 (18,7)**	**4.635 (19,7)**	**2.087 (8,9)**	**628 (2,7)**

Nota: Letras diferentes (a, b) indicam diferenças significativas entre as regiões.

A Figura 2 representa as dificuldades relatadas na obtenção de medicamentos e no alcance do tempo semanal de tratamento hemodialítico ≥ 12h para o manejo dos pacientes, e a Figura 3 mostra essas dificuldades por região/medicamento. Calcitriol, paricalcitol, cloridrato de cinacalcete e cloridrato de sevelâmer foram relatados como de fácil obtenção (de nunca a raramente com dificuldade). De maneira preocupante, 14 a 23% das unidades relataram dificuldades para obtê-los, destacando as diferenças na forma como essas dificuldades são manejadas. Uma observação crucial dos dados são as dificuldades relatada por quase 40% das instalações em obter sais de cálcio; além disso, cerca de 30% delas também indicaram dificuldades em prescrever hemodiálise ≥12 horas por semana e em obter desferrioxamina para tratamento de intoxicação por alumínio. Por fim, a maior dificuldade relatada por quase metade das unidades foi na obtenção de tiossulfato de sódio para o manejo da calcifilaxia. Vale ressaltar que, com exceção do tiossulfato de sódio, todos esses medicamentos são fornecidos pelo SUS.

**Figura 2:** Dificuldades na obtenção de medicamentos e na prescrição de hemodiálise para o manejo desses indivíduos; as legendas são apresentadas no sentido horário.

**Figura 3:** Dados classificados por região. Cada ponto representa uma unidade respondente e a linha de corte no nível “três” da escala Likert (“raramente”) é indicada por uma linha tracejada.

Analisados por região (Figura 3), calcitriol, paricalcitol, cloridrato de cinacalcete, cloridrato de sevelâmer e tiossulfato de sódio foram declarados como sendo, em média, “frequentemente” a “sempre” difíceis de obter na região Norte, enquanto os sais de cálcio e a desferrioxamina foram igualmente difíceis de obter na região Nordeste. A programação de mais de 12 horas de hemodiálise por semana foi declarada como sendo, em média, “frequentemente” a “sempre” viável na região Sul.

Os dados referentes às opções de encaminhamento para a PTx, o tempo de espera e os principais obstáculos à realização da PTx estão representados graficamente na Figura 4. As três principais opções de encaminhamento para a PTx são os serviços públicos com equipe especializada em DMO-DRC, seguidos pelos hospitais do SUS e hospitais conveniados. Preocupantemente, o tempo de espera para o procedimento foi, em sua maioria, superior a dois anos e raramente realizado com menos de seis meses de espera; além disso, a escassez de cirurgiões de cabeça e pescoço e a falta de leitos de enfermaria para internações foram declaradas como obstáculos importantes. Dificuldades na autorização do procedimento no âmbito da operadora do contrato do plano de saúde representaram cerca de 27% das dificuldades. Além disso, os exames pré-operatórios e a obtenção de medicamentos para o manejo pós-operatório foram apontados como barreiras. A falta de possibilidade de encaminhamento representa 10%.

**Figura 4:** Respostas sobre as opções de encaminhamento para paratireoidectomia (PTx), tempo médio de espera e principais obstáculos para a realização da PTx; as legendas são apresentadas no sentido horário.

A comparação entre os dados do estudo de 2011 e esta atualização de 2024 é apresentada na Tabela 2. Embora uma comparação estatística direta esteja além do objetivo da presente atualização, o número absoluto - e o percentual - de pacientes com PTH > 1.000 pg/mL e aqueles com indicação ou que foram submetidos a PTx permaneceram semelhantes, representando uma pequena melhora nos últimos treze anos de manejo dessa condição.

**Tabela 2:** Comparação entre dados selecionados da pesquisa de 2011^33^ e a atualização de 2024

Estudo	Número total de indivíduos	Número total (%) de pacientes com PTH > 1.000 pg/ml	Unidades (%) com tempo de espera de 12 a 24 meses para PTx	Unidades (%) que declararam escassez de cirurgiões de cabeça e pescoço
2011	32.264	3.463 (10,7)	19 (8)	64 (28)
2024	23.535	2.087 (8,9)	29 (25)	63 (18)

## Discussão

Este estudo teve como objetivo atualizar e aprofundar o levantamento de 2011^33^. A taxa de prevalência de HPTS grave foi relatada em cerca de 9%, considerando níveis de PTH > 1.000 pg/mL, uma situação que requer PTx, podendo atingir 20% considerando níveis séricos de PTH > 600 pg/mL. Evidências adicionais de que a intervenção cirúrgica é a melhor opção de tratamento foram relatadas recentemente^16^ e indicadas para pacientes com DRC em diálise^17–20^. Se fosse adotado o ponto de corte de 800 pg/mL, conforme recomendado pelas Diretrizes Brasileiras de Prática Clínica para DMO-DRC, a prevalência de HPTS seria maior. Vale ressaltar também que uma porcentagem significativa de indivíduos com PTH de até 500 pg/mL tampouco responde totalmente à terapia medicamentosa^34^, o que aumentaria ainda mais o número de indivíduos que necessitam da cirurgia de PTx. É notável que o número de pacientes submetidos à PTx represente 2,7% dos indivíduos incluídos, algo que não foi relatado na pesquisa anterior. Em nossa opinião, essa informação é preocupante. Primeiramente, é necessário enfatizar que medicamentos, mesmo os mais simples, como sais de cálcio, e condições adequadas para a prescrição do tempo apropriado de terapia dialítica são maneiras eficientes e de baixo custo para prevenir e retardar a progressão da DMO-DRC. Superar esses obstáculos é um elemento essencial para mudar essa realidade.

Por ser um procedimento cirúrgico de complexidade moderada, a PTx pode ser realizada em hospitais de atenção secundária que contam com uma equipe de nefrologistas (suporte à diálise) e cirurgiões de cabeça e pescoço^33^. Nos resultados de 2024, os dois principais obstáculos - poucas unidades especializadas em DMO-DRC que apoiem esse procedimento e a escassez de cirurgiões de cabeça e pescoço – permanecem como desafios significativos. No entanto, os nefrologistas estão plenamente capacitados para o manejo de pacientes com DMO-DRC.

Cerca de 90% dos pacientes submetidos à PTx desenvolvem a síndrome da fome óssea durante o período pós-operatório^35^, exigindo quantidades elevadas de sais de cálcio e calcitriol por várias semanas. Embora a maioria das unidades receba esses medicamentos do Programa de Medicamentos Especiais do SUS, restrições burocráticas ainda podem causar atrasos na dispensação de quantidades insuficientes. Esse problema foi relatado como “frequente” por 17,2% das unidades para sais de cálcio e 8,6% para calcitriol. Consequentemente, a gestão da unidade pode precisar redirecionar esses medicamentos de pacientes com hipercalcemia e/ou hiperfosfatemia.

De acordo com a Demografia Médica no Brasil 2023 (https://amb.org.br/wp-content/uploads/2023/02/DemografiaMedica2023_8fev-1.pdf), o número de cirurgiões de cabeça e pescoço aumentou para 1.403 profissionais. A maioria reside em São Paulo, conforme indicado anteriormente^33^ e talvez a preocupação crucial em relação aos desafios técnicos, políticos, econômicos e organizacionais que dificultam a colaboração entre gestores de saúde pública, unidades de diálise, nefrologistas, cirurgiões de cabeça e pescoço e centros que realizam PTx ainda seja relevante em 2024.

O obstáculo anteriormente relatado quanto aos exames pré-operatórios (~39%)^33^ para a realização de PTx não parece ser a principal questão em ٢٠٢٤, conforme indicado por menos de ٥٪ dos respondentes nesta atualização. A concentração observada de unidades na região sudeste pode ter influenciado essa associação. Em contrapartida, em 2024, o obstáculo declarado de poucas unidades especializadas em DMO-DRC que apoiem a PTx, associado à escassez declarada de cirurgiões de cabeça e pescoço, parecem ter sido os dois principais problemas. A falta de unidades de terapia intensiva e leitos de enfermaria também foi citada como um problema, assim como em 2011^33^.

Essa atualização da pesquisa apresenta limitações. Embora o objetivo deste estudo tenha sido atualizar a pesquisa de 2011^33^, a taxa de resposta foi significativamente menor, com 1,9 vezes mais unidades de diálise (226 naquele estudo, incluindo 32.264 indivíduos) participando da pesquisa anterior. Como resultado, os dados aqui apresentados devem ser interpretados com cautela, especialmente considerando que o número de respondentes foi reduzido para 114, sendo a maioria da cidade de São Paulo. Este censo não teve como objetivo analisar as causas e os fatores envolvidos nas dificuldades relacionadas ao acesso ao tratamento clínico e cirúrgico do HPTS. Outro aspecto que chama a atenção é que, em 2011, o número médio de pacientes era de 143 por centro de diálise e, em 2024, esse número subiu para 206 por centro, um aumento de 44%. O impacto desse aumento na DMO-DRC não pôde ser captado por esta pesquisa, mas merece ser considerado como um possível fator associado aos resultados observados.

Embora o questionário não tenha se concentrado na continuidade do fornecimento de medicamentos, é razoável especular que interrupções na medicação possam desempenhar um papel negativo no controle do HPTS. Finalmente, este estudo fornece uma pesquisa nacional com dados representativos da população em diálise em todas as regiões do país e revela coletivamente os desafios no acesso ao tratamento clínico e cirúrgico do HPTS.

## Conclusão

Alguns aspectos do HPTS em pacientes em diálise melhoraram desde 2011. No entanto, a doença permanece altamente prevalente no Brasil, e um número significativo de indivíduos não tem acesso à PTx ou precisa esperar um longo período por esse procedimento cirúrgico, enquanto enfrentam dificuldades substanciais para obter tratamento clínico. Embora os esforços do governo para fornecer acesso a tratamento clínico e cirúrgico aos pacientes sejam louváveis, pode haver espaço para melhorias no sistema. Espera-se que esses dados possam potencialmente apoiar melhorias no sistema de saúde para esses pacientes por meio de um melhor atendimento público. Uma colaboração mais eficiente entre nefrologistas e cirurgiões, e entre os prestadores de serviços de saúde pública e privada, é urgentemente necessária para mudar essa realidade.

## Data Availability

Todos os dados gerados ou analisados durante este estudo estão incluídos neste artigo.
